# Improving Speed and Efficiency of DESI Imaging with the Xevo MRT Mass Spectrometer for Analyte Mapping

**DOI:** 10.3390/metabo16060429

**Published:** 2026-06-18

**Authors:** Mark Towers, Emmanuelle Claude, Lisa Towers, Helen Yates, Joanne Ballantyne

**Affiliations:** Waters Corporation, Wilmslow SK9 4AX, UK; emmanuelle_claude@waters.com (E.C.); lisa_towers@waters.com (L.T.); helen_yates@waters.com (H.Y.); joanne_ballantyne@waters.com (J.B.)

**Keywords:** DESI, imaging, Xevo MRT MS, metabolites, lipidomics

## Abstract

**Background:** Recent technology improvements have enabled desorption electrospray ionisation (DESI) mass spectrometry imaging to achieve down to 5 µm (pixel) image resolution. However, operating at this resolution introduces challenges, particularly regarding increased total analysis time and the need for sufficient instrument sensitivity to detect analytes from very small tissue areas. **Methods:** High mass and image resolution DESI imaging was performed on rat brain tissue using a Xevo™ MRT benchtop mass spectrometer equipped with a multi-reflecting time-of-flight mass analyser and a DESI XS source. Data acquisition was conducted at speeds of up to 100 Hz. Sensitivity was assessed using a dilution series of five Active Pharmaceutical Ingredients (APIs) spotted onto porcine liver tissue. Signal detection limits were evaluated using extracted ion chromatograms (XICs) with signal-to-noise (S/N) calculations against blank samples. Additionally, enhanced duty cycle (EDC) was applied to evaluate improvements in analyte signal intensity across specific mass ranges in both positive and negative ionisation modes. **Results:** At acquisition speeds of up to 100 Hz, excellent data quality was achieved, with signal intensity remaining suitable for analytical applications. All five tested APIs were detectable at concentrations of 25 pg/mm^2^. Three of the five compounds were further detected at concentrations as low as 2.5 pg/mm², with signal-to-noise ratios greater than 5. The application of EDC resulted in a significant increase in analyte signal intensity within the targeted mass ranges, particularly for small molecule endogenous metabolites and lipids, in both ionisation modes. Furthermore, the system demonstrated substantially improved spectral quality, achieving mass resolution up to 100,000 FWHM. This enabled the resolution of previously indistinguishable analytes with significantly improved mass accuracy compared to systems operating at approximately 30,000 FWHM. **Conclusions:** The Xevo™ MRT mass spectrometer with DESI XS source enables high-resolution DESI imaging at speeds up to 100 Hz without compromising data quality or sensitivity. The system demonstrates excellent detection limits for pharmaceutical compounds and improved performance through enhanced duty cycle operation. Overall, the combination of high spatial resolution, increased mass resolution, and improved spectral quality allows for more accurate analyte differentiation, representing a significant advancement over lower-resolution systems.

## 1. Introduction

DESI is a surface analysis technique first introduced in 2004 [1]. It has subsequently become well established for mass spectrometry imaging applications, allowing the spatial mapping of molecules from a substrate. DESI analysis requires no pretreatment of the sample and allows for the analysis of a wide variety of molecules [2,3,4,5,6]. The principle behind DESI is based upon electrospray ionisation: a fine stream of primary charged and solvent droplets is sprayed towards the sample surface, creating a small pool of solvent which desorbs the analytes present. Additional spray then interacts with the pool, causing secondary droplets—containing the analytes—to be ejected from the pool and into the mass spectrometer inlet for mass measurement. In DESI imaging, the sample is rastered under the sprayer and positional information is recorded for each scan/spectra generated. These spectra form pixels which when combined with the positional information allow the creation of an image that maps the distribution of analytes within the sample. With advances in DESI source technology [7,8,9], there has been a drive towards higher spatial resolution [5,10,11] and it has recently become possible to perform image acquisitions with a pixel size as low as 5 µm [10], giving users the ability to spatially resolve and explore smaller features than ever before. This can pose potential challenges for some of the many imaging applications DESI is utilised for [5,11,12,13,14,15,16], as often these biological or chemical questions require acquisition of large image areas. When acquiring data with high image resolution, with every reduction in pixel size the image acquisition time exponentially increases. For example, an image on an area of tissue 1 mm squared imaged at 50 µm pixel size with a 10 Hz/0.1 s scan speed would take ~40 min acquisition time. Decrease the pixel size to 5 µm and the acquisition time increases to ~4000 min, or ~66 h with the same scan speed of 10 Hz/0.1 s. Secondly, with reduction in pixel size the area of tissue desorbed during any one scan is exponentially reduced and with it, the signal intensity. Acquisition time can be improved by increasing instrument scan speed, but this also has a significant impact on signal response. As with DESI, the overall signal response is roughly proportional to the time scanning per pixel. Areas targeted for increased spatial resolution can be minimised by utilising the microscope mode analysis design [10], whereby an image is acquired with a large pixel size and small sub-sections of the tissue are re-imaged with the high resolution pixel size, thanks to the non-destructive nature of DESI [17].

Here, we demonstrate high resolution DESI imaging with high mass and image resolution on a benchtop mass spectrometer: a Xevo™ MRT mass spectrometer fitted with a DESI XS source. The improved ion optics and multi-reflecting time-of-flight (ToF) technology produces a mass resolution of up to 100,000 FWHM [18] (full width half maximum), independent of acquisition speed.

Sensitivity is increased through ion optic improvements refocussing the ion beam after the collision cell dispersion, shaping it with a ~5× increased diameter and significantly reducing the angular divergence of the ions. Utilising two sets of deflectors to ensure beam alignment and a heated collimator to remove beam wings, these new ion optics allow better conformation/collimation of the ion beam, optimising ion passage into the orthogonal accelerator (OA), and maintain a high transmission efficiency of greater than 90% (for currents up to 1 nA). These improvements reduce ion losses and dispersal, maximising analyte signal strength and mass resolution.

Advanced ion handling and electronic architecture developed for this mass spectrometer system has minimised the duty cycle and inter-scan delay to 2 ms, enabling a significantly improved data collection time per scan cycle, allowing for much faster scan speeds. The orthogonal accelerator has an improved elongated design, allowing for tightly focused packets of ions (of up to 22 mm) to be formed from the continuous ion beam. This increase in packet size and optimised ion handling reduces the duty cycle and improves ion transmission through the flight tube. The upgraded electronics means voltages settle faster, electronic shielding is enhanced and the RF (radio frequency) has been redesigned to enhance ion transmission. These improvements combined help to remove inter-scan crosstalk, enabling a shorter fixed inter-scan delay period and improved data scheduling, meaning the mass spectrometer can scan faster. Along with hardware and firmware improvements, there is also an option for further duty cycle enhancement for specific mass ranges. Enhanced duty cycle (EDC) mode optimises the pusher frequency and transfer of ion packets into the flight tube to improve the transmission of ions around a certain mass range, boosting their signal. With EDC, a portion of the mass range signal intensity can be enhanced, with the sacrifice of sensitivity from outside the selected range, allowing for a semi-targeted mode of acquisition for which the window of the enhancement varies depending on the targeted mass range.

In this paper we explore the applicability of this instrument to the performance of DESI imaging, specifically investigating whether DESI imaging experiments can be successfully performed at high acquisition rates, whilst still maintaining image clarity and achieving relevant analyte coverage. We also look at how instrument sensitivity for both endogenous metabolites/lipids or APIs from tissues can be improved through the use of EDC. The improved ion handling, sharpening of spectral peaks, improved data handling and the option for features such as EDC mode, gives an improved sensitivity and mass accuracy compared to other DESI-capable time-of-flight instruments [10]. This paper shows that the spectral and image quality can be maintained even at scan speeds of up to 100 Hz. In addition, the improved spectral peak shape and mass resolution allow for isoforms and other compounds with similar molecular weight to be separated, providing a spectrum with more detected analytes than instruments with lower mass resolution, and provide a higher confidence in mass and analyte isotopic distribution-based identifications.

## 2. Materials and Methods

Rat brain tissues were sectioned at 18 µm. A drug dilution series, propranolol, terfenidine, moxifloxacin, reserpine and chloroquine (all APIs were aquired from Merck Sigma, Poole, UK) at concentrations 10 pg, 100 pg, 1000 pg and 10,000 pg, was prepared in 50:50 MeOH:water (Merck Sigma, Poole, UK). 1 µL of each solution was spotted onto an 18 µm thick porcine liver section as a complex matrix, along with a blank 50:50 MeOH:water.

DESI imaging was performed on a Xevo™ MRT MS (Waters, Wilmslow, UK) using novel Waters MS Imaging software within a waters_connect™ environment. Data were collected at acquisition speeds from 5 to 100 Hz, in both untargeted and semi-targeted (EDC) acquisition modes. An ACQUITY M-class µBSM (Waters, Milford, MA, USA) with a DESI low-flow capability with back pressure regulator kit (Waters, Wilmslow, UK), provided <250 nL/min solvent flow for high resolution imaging and 2 µL/min for lower resolution imaging, solvent composition 95:5 MeOH:Water (LCMS grade solvent) ). DESI settings were: heated inlet capillary at room temperature (RT) for the APIs and small metabolites experiments, 250 °C for the lipid experiments in positive ionisation mode and 450 °C for the lipid experiments in negative ionisation mode. Sprayer positioning was optimised for each experiment following the guidance described by Towers et al. [4]. For high resolution imaging with pixel sizes of 5–20 µm the sprayer was moved as close to the surface as possible < 1 mm. For lower resolution imaging with pixel sizes of 25 and 50 µm the sprayer height was optimised for maximum signal, typically in the range of 2–5 mm (pixel sizes are stated in indervidual figure legends). Capillary voltages were optimised in the range of 0.6–0.8 kV, the sprayer angle was fixed at 70°, the inlet height was set to >1 mm and the sprayer-to-inlet distance was optimised on an experiment-per-experiment basis to maximise signal, typically 2 to 8 mm with a nebulising gas pressure of 1–1.35 bar.

Semi-targeted EDC mode was performed with the target mass of 80 Da, 150 Da, 225 Da, 300 Da, and 800 Da in negative ionisation mode and 800 Da in positive ionisation mode.

Data were processed within the Waters High-Definition-Imaging™ software v1.9 and Waters MS Imaging software v1.0 within waters_connect software v4.2. Where stated in the figure legend the data was lock mass corrected using continuous lock mass correction (CLMC). Either the internal lipid at *m*/*z* 834.52906 putatively identified as phosphatidylserine (PS) 40:6 was used as the lock mass, with a correction period of 20 min and a collection period of 5 min or the internal lipid at *m*/*z* 885.54985 putatively identified as phosphatidylinositol (PI) 38:4 was used, with a correction period of 1 min and a collection period of 0.1 min. No other data was lock mass corrected.

For image creation in HDI, the MS resolution was set to 80,000 FWHM and the ion extraction window was set to 0.01 Da, and the top 1000 ion images by sum intensity were extracted.

XICs and s/n ratio calculations were processed in the Toolkit app within the waters_connect software v4.2.

## 3. Results and Discussion

**Figure 1:**
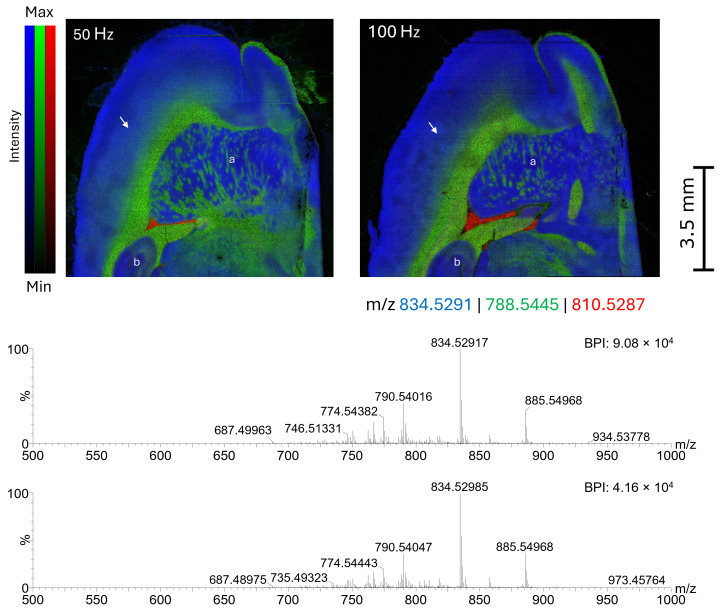
High signal intensity at high scan speeds—RGB lipid ion images (10 µm pixel size) acquired at 50 Hz (**left**); and 100 Hz (**right**), Red: PS 38:4, Blue: PS 40:6, Green: PS 36:1. MS spectra acquired at 50 Hz (**top**) and 100 Hz (**bottom**) of the lipid mass range in negative mode, BPI is stated for randomly selected pixels on the spectra (indicated by arrows) in the isocortex. The Caudoputamen (a) and Hippocampal formation (b) have been highlighted as regions used for visual inspection. The data was lock mass corrected using the *m/z* 834.52906 peak. Images normalised to total ion count (TIC).

Sections of rat brain were analysed on a Xevo MRT mass spectrometer (MS) fitted with a DESI XS source at differing acquisition speeds. Previously, increasing the scan rate of imaging data acquisition led to a loss of sensitivity with fewer analytes being detected. However, with the Xevo MRT MS we can demonstrate (Figure 1) that even with speeds of 50 or 100 Hz in MS mode, whilst a proportional decrease in signal is observed, the data quality is still excellent (mid e4) and signal intensity (particularly in the lipid region) remains suitable for most applications.

**Figure 2:**
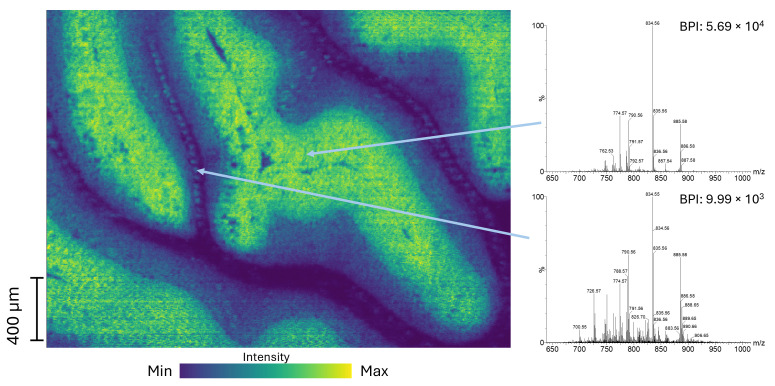
A 5 µm pixel size positive mode image of *m*/*z* 834.53 in negative mode from a sub-section of rat brain acquired at 50 Hz with EDC, upper spectrum a single pixel spectrum from a high abundance region, lower spectrum and single pixel spectrum from an individual Purkinje cell. The data was lock mass corrected using the *m/z* 834.52906 peak.

The images in Figure 1 show two consecutive rat brain sections comprising three overlayed lipid species putatively identified as phosphatidylserine (PS) 40:6 (lock mass peak) (-47 ppb), PS 36:1 (-266 ppb) and PS 38:4 (-444 ppb) demonstrating sub-500 ppb mass accuracy, using a red, green, blue (RGB) overlay. It can be seen that each lipid has a different localisation within the tissue. When the scan speed is increased from 50 Hz to 100 Hz, there is no apparent loss in image fidelity or feature clarity, and the two images visually appear comparable. Examining the regions within the images (Caudoputamen and Hippocampal formation), we can see that the size of the features present match, indicating no change in apparent resolution. Beneath the ion images are spectra from a single scan, and again it can be seen that there is a similar number of analytes detected and the spectral pattern is closely matched between the two scan speeds. To further assess the spectral similarity, the two data sets were aligned using the build target list functionality in HDI. The 1000 features extracted from the data were compared and if the mass was within 0.0002 Da, the masses were averaged. If a mass was only found in one feature set, it was added to the target list. This resulted in a list of 1265 total features from both data sets combined, and the data was reprocessed using this target list. Five ROIs of 100 pixels were randomly extracted from the isocortex of each tissue, to compare the Pearson product moment coefficient was calculated (Supplemental Data S1). The average intra-correlation for the 50 Hz data was found to be R 0.995 with a standard deviation of 0.005 (R^2^ = 0.991). For the 100 Hz data this was found to be R 0.987 with a standard deviation of 0.011 (R^2^ = 0.974). The average inter-correlation between the 50 Hz and 100 Hz was found to be R 0.989 with a standard deviation of 0.008 (R^2^ = 0.978), showing that the data from similar regions of the two data sets correlated very highly, although with potentially slightly more variance within the 100 Hz sample. Both images were acquired with a pixel size of just 10 µm, allowing visualisation of fine brain structures and features. By using the reduced flow rate of 250 nL, the pixel size can be further reduced to allow for high resolution imaging with a 5 µm pixel size. Figure 2 shows an ion image of the *m*/*z* 834.53 peak putatively identified as PS(40:6). Within the image, Purkinje cells are clearly visible. Individual spectra from single pixels had been extracted, allowing the display of a spectrum from a single Purkinje cell and the single pixel spectrum from the Cerebellum. The Purkinje cells have been calculated to be approximately 4–5 pixels in size which closely matches the reported size in adult rats of approximately 22–23 µm [19] demonstrating sub-10 µm resolution.

**Figure 3:**
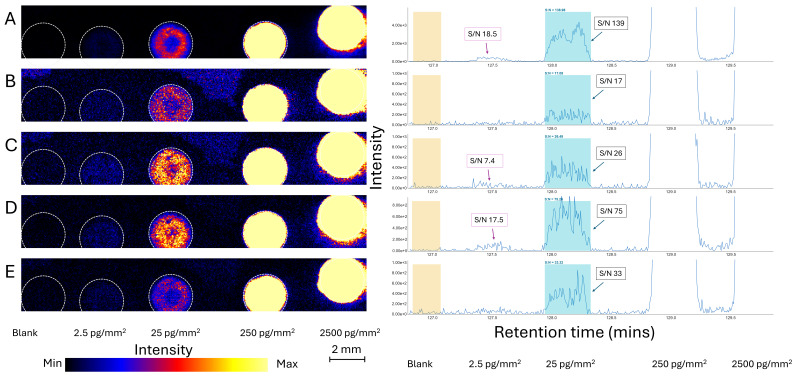
Sensitivity of small molecule drugs in positive mode on tissue: API compounds: (**A**) terfenadine (*m*/*z* 472.3), (**B**) moxifloxacin (*m*/*z* 402.2), (**C**) chloroquine (*m*/*z* 320.2), (**D**) propranolol (*m*/*z* 260.1), (**E**) reserpine (*m*/*z* 609.3) acquired in full scan MS mode at 2 Hz. (**Left**) Ion images for each API dilution series. (**Right**) Concentration curve of 2.5 pg/mm^2^ to 2500 pg/mm^2^ on tissue, the signal to noise is calculated on the 25 pg/mm^2^ on-tissue spot and indicated for the 2.5 pg/mm^2^ spots when S/N > 5. Data aquired with a 50 µm pixel size.

To investigate system sensitivity, an API dilution series was prepared containing five commercially available drugs: terfenidine, moxifloxacin, chloroquine, propranolol and reserpine. To be cautious, a S/N threshold of 5 was used to determine detection which is widely accepted for LC/MS data for quantitation, with a S/N to noise of 3 normally used for detection. The dilution series covered four orders of magnitude when 1 µL was spotted on tissue, 10 pg, 100 pg, 1000 pg and 10,000 pg, and included a solvent blank. Each spot covers an area of approximately 4 mm^2^ giving an approximate on-tissue concentration of 2.5 pg/mm^2^, 25 pg/mm^2^, 250 pg/mm^2^, and 2500 pg/mm^2^, respectively. Figure 3 shows the ion images and extracted ion chromatograms acquired from each of the four analyses. When these API spots were analysed in conventional full (50–1200 Da) scan MS mode, with 50 µm pixel sizes at 2 Hz, it can be seen that each drug is clearly visible in the ion images and extracted ion chromatograms (XIC) at 25 pg/mm^2^ (see coloured with a light blue on the XICs).

It also shows trace amounts for three drugs spotted on the tissue at the 2.5 pg/mm^2^ concentration. The RMS signal to noise ratio has been calculated on the XICs for the 2.5 pg/mm^2^ and 25 pg/mm^2^ concentration of each drug using the blank spot location as representative of noise. All compounds have a signal to noise greater than 5 at 25 pg/mm^2^ concentration and three (terfenadine, chloroquine and propranolol) have a signal to noise greater than 5 at 2.5 pg/mm^2^ concentration.

**Figure 4:**
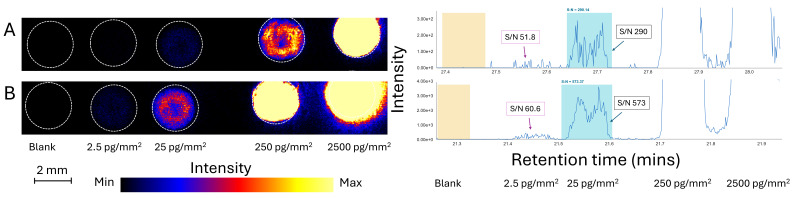
Comparison of the (**A**) terfenadine product ion image *m*/*z* 436.3 standard duty cycle MS/MS mode vs. (**B**) terfenadine product ion image *m*/*z* 436.3 semi-targeted MS/MS EDC *m*/*z* 436.3. (**Left**) Ion images of the terfenadine product ion *m*/*z* 436.3 dilution series in positive mode. (**Right**) XIC of the terfenadine product ion *m*/*z* 436.3 dilution series of 2.5 pg/mm^2^ to 2500 pg/mm^2^. Data aquired with a 50 µm pixel size.

When a user application has a known target class of compounds, it is possible to further increase the mass spectrometer sensitivity for target mass ranges using EDC. The width of the enhancement band is proportional to the target central mass, with lower target masses having a smaller enhancement window, for example, the window for enhancement at *m*/*z* 150 is typically around 100 Da, and at *m*/*z* 700 is around 300 Da wide, i.e., +/− 150 Da from the set mass. One potential semi-targeted application using EDC is for the evaluation of the distribution of an API within an animal model (whole body or organ) to ascertain if the API is getting to the right target organ. The maximum specificity and sensitivity can be achieved using a MS/MS experiment, whereby the precursor *m*/*z* of the API is selected within the quadrupole then collision induced disassociation (CID) is applied to produce and subsequently map one of the product ions of the API. Using this approach, the dilution series was imaged with 50 µm pixel size at 10 Hz in MS/MS mode choosing *m*/*z* 472.3 for the terfenadine precursor, and collision energy was optimised for maximal signal intensity of its product ion *m*/*z* 436.3. Then to further enhance the sensitivity, the same analysis was performed with the EDC set for *m*/*z* 436.3. Figure 4 displays the ion images (same intensity scale used) and XIC comparing *m*/*z* 436.3 in MS/MS mode (A) with *m*/*z* 436.3 (product ion of terfenadine) in MS/MS mode with EDC *m*/*z* 436.3 (B). In the data acquired with the semi-targeted MS/MS EDC, we see an improved ion image intensity and a higher signal to noise ratio than without the EDC active.

**Figure 5:**
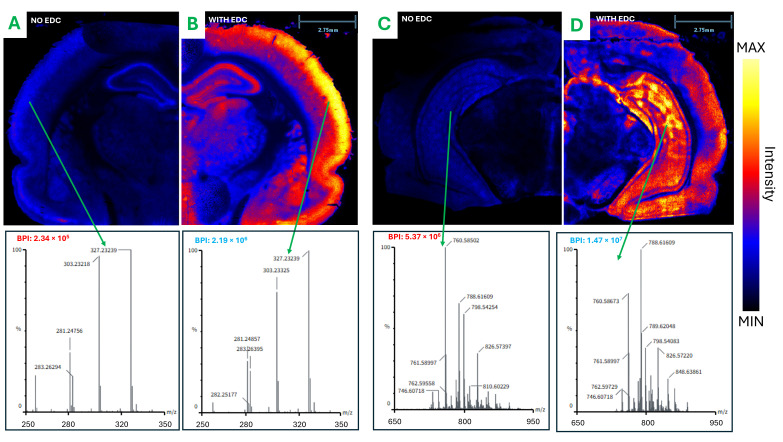
A section of rat brain acquired in negative ionisation mode: the left half with (**A**) no EDC active using a typical 50–1200 Da scan window. The right half (**B**) acquired with EDC on and targeting a small molecule metabolites portion of the spectrum (250–350 Da). Images generated from analytes with *m*/*z* 327.2324 Da, putatively identified as DHA, and the intensity scales are the same for both halves of the brain. A section of rat brain acquired in positive ionisation mode: the right half (**C**) acquired with no EDC active using a typical 50–1200 Da scan window. The left half (**D**) acquired with EDC on and targeting the lipid portion of the spectrum (700–900 Da). Images generated from analytes with *m*/*z* 837.6691 Da, putatively identified as PS O-39:0, and the intensity scales are the same for both halves of the brain. Spectra formed from 100 combined pixels taken indicated by arrow, shown below each image. Images are not normalised. Data aquired with a 25 µm pixel size.

A second application where EDC can be useful is for the detection of low abundant small endogenous metabolites. EDC was applied during the imaging of rat brains, analysis was performed with both low target mass range and a lipid target mass range. Figure 5 shows the improvement in sensitivity for the applied target mass ranges; acquisition was performed at a pixel size of 25 µm at 10 Hz.

A coronal section of rat brain was analysed in negative ionisation mode with no EDC (Figure 5A), and with EDC set with target mass of 300 Da (Figure 5B), giving an approximate enhancement mass window of 250–350 Da. Figure 5A,B show negative ionisation mode images of analyte 327.2324, putatively identified as docosahexaenoic acid (DHA), with a 1.68 ppm mass error. It can be seen visually that the intensity of the analyte is much stronger within the EDC acquisition when the intensity scale is identical between the two images, and that the overall spectral intensity of the analytes is higher for the EDC acquisition. A coronal section of rat brain was also analysed in positive ionisation mode with no EDC (Figure 5C), and with EDC set with target mass of 800 Da (Figure 5D), giving an approximate enhancement mass window of 700–900 Da. Figure 5C,D show positive ionisation mode images of analyte 837.6691, putatively identified as PS O-39:0 with a 0 ppm mass error. As with the negative mode acquisition, the intensity of the analyte is much stronger within the EDC acquisition with the intensity scale identical between the two images, and the overall spectral intensity of the analytes is also higher in the EDC acquisition.

**Figure 6:**
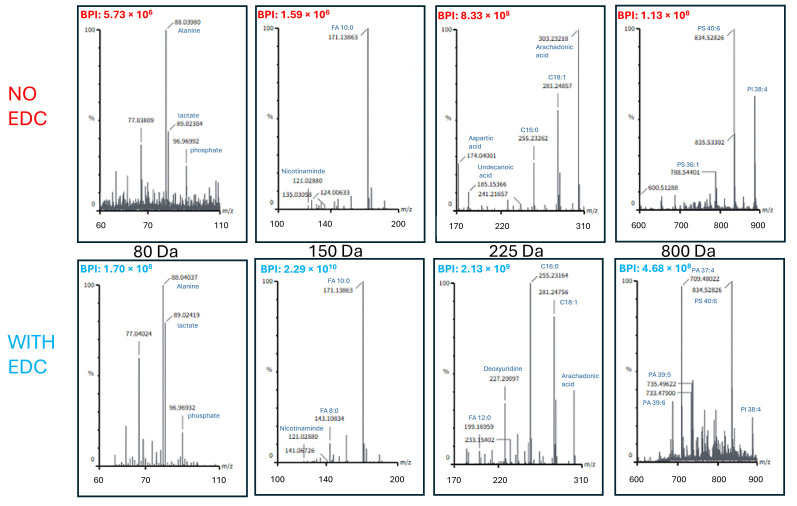
Zoomed spectral regions taken from a 5000-pixel section of a rat brain (imaged at 25 µm pixel size), acquired in negative ionisation mode with no EDC active—using a typical 50–1200 Da scan window (**top spectra**), and EDC set to 80 Da, 150 Da, 225 Da, and 800 Da (**bottom spectra**). Some key metabolites and lipids are highlighted on each spectrum, highlighting the increase in signal between 4 and 30 times, molecule-dependent.

As part of the above, with and without EDC brain acquisitions, areas of coronal rat brain sections were also analysed (with a 25 µm pixel size) in negative ionisation mode: with EDC set with target mass of 80 Da, 150 Da, 225 Da, and 800 Da, and EDC not active. A 5000-pixel region of interest was drawn for each of these acquisitions transecting various brain regions. Figure 6 shows spectra from each of these analyses alongside the respective mass range from a no EDC applied, i.e., standard duty cycle—typical 50–1200 Da mass range acquisition.

The improvements in analyte signal for each of these spectral regions is demonstrated. We have highlighted a few key metabolites displayed in the spectra, these are: alanine, lactate, phosphate, nicotinamide, fatty acid (FA) 8:0, FA 10:0, FA 12:0, deoxyuridine, palmitic acid (FA 16:0), oleic acid (FA 18:1), arachidonic acid (FA 20:4), phosphatidic acid (PA) 39:6, PA 39:5, PA 37:4, PS 40:6 and PI 38:4. There is a significant increase in the analyte signals when EDC was utilised over both the small molecule and the lipid mass ranges compared to a typical standard duty cycle 50–1200 Da scan window. This approach yields between a 4- and 30-fold increase in signal, molecule-dependent. It should be noted that this technique is described as semi-targeted, as the spectral regions outside of the enhancement window will see a reduction in signal intensity compared to an analysis not utilising EDC mode. This means that only the spectral region within the enhancement window will see an improvement in signal and the rest of the spectra will see a decrease in signal compared to non-EDC acquisitions.

**Figure 7:**
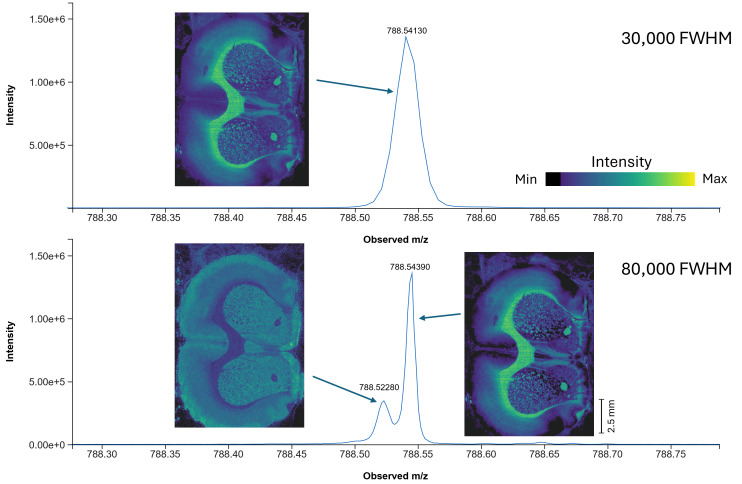
Improved mass resolution with the Xevo MRT compared to other benchtop mass spectrometers, giving higher number of analytes detected and better mass accuracy for putative IDs. The figure shows differential distribution from a coronal rat brain acquired with an 80,000 FWHM mass resolution in positive mode with a 20 µm pixel size at 50 Hz, highlighting the presence of two lipid ion images vs. a single ion image acquired with the mass resolution of 30,000 FWHM. The data was lock mass corrected using the *m*/*z* 885.54985 Images are not normalised.

Other than increased sensitivity, the Xevo MRT MS provides a user with up to 100,000 FWHM mass resolution on a benchtop mass spectrometer; other benchtop mass spectrometers available for DESI imaging applications provide around 30,000 FWHM mass resolution. This significant increase in mass resolution enables the instrument to detect a greater number of analytes, pulling apart isomers and compounds with very tight mass separation. This means that two (or more) analytes not separated by mass with a 30,000 FWHM mass spectrometer can be separated and accurately identified using the Xevo MRT MS. Figure 7 shows one such example, acquired at a pixel size of 20 µm at 50 Hz.

When the brain tissue was analysed by a conventional benchtop time-of-flight mass spectrometer there appears to be a single analyte. When this is displayed as an image it appears as though this analyte is present across the whole tissue. The single spectral peak—with the peak top mass of 788.5413 Da—is putatively identified as protonated PS 36:2 (C42H78NO10P) with a −2.9 ppm mass error. However, when this same brain was analysed by the Xevo MRT MS, the increased mass resolution identified two distinct analytes within the 788.5 Da region of the spectra (the area covered by the single peak at 30,000 FWHM mass resolution). One analyte at 788.5228 Da putatively identified as a protonated phosphatidylethanolamine (PE) 40:8 (C_45_H_74_NO_8_P) with a −0.38 ppm mass accuracy, and one analyte at 788.5439 Da putatively identified as protonated PS 36:2 (C_42_H_78_NO_10_P) with a 0.38 ppm mass error. When these two lipids are displayed as an overlayed image, they demonstrate significantly different localizations within the tissue. The PE 40:8 lipid is predominantly localised to the corpus colostrum and anterior commissure, with some speckling within the corpus striatum, whereas PS 36:2 appears to be found quite evenly distributed throughout the corpus striatum and cortex.

## 4. Conclusions

Here, we have described the use of a Xevo MRT mass spectrometer to advance DESI imaging capabilities, providing solutions to traditional analysis constraints and therefore pushing the boundaries of current MS Imaging. This new system delivers improved analysis speeds of up to 100 Hz and—with EDC—maintains sufficient spectral and image quality for most applications, compared to previous generation mass spectrometers capable of a maximum of 30 Hz scan speeds, often with a reduction in spectral and image quality compared to slower speeds.

Utilising the EDC mode, whereby pusher ion transfer and frequency are optimised for a set target mass, the detection of ions within the target range can be increased between 4 and 30×, molecule-dependent. Although the use of EDC does decrease the available mass range, it is still possible to acquire large portions of the lipid or small molecule regions. The sensitivity increase in this semi-targeted acquisition mode facilitates the high speed and high resolution imaging without compromising the quality of the data acquired.

Sensitivity of small molecule APIs has been demonstrated with conventional full scan analysis down to 2.5 pg/mm^2^ and 25 pg/mm^2^ on tissue, with all five API compounds showing a signal to noise ratio >5 at 25 pg/mm^2^ and three of the API compounds showing a signal to noise ratio >5 at 2.5 pg/mm^2^. The signal to noise ratios can be further improved using MS/MS and MS/MS with EDC for a pseudo ToF MRM experiment. In our example, we saw an improvement in the signal to noise for terfenadine from 18.5 (MS mode) to 51.8 (MS/MS) and 61.6 (MS/MS with EDC) for the 2.5 pg/mm sample. Lastly, we show that by delivering a mass resolution of up to 100,000 FWHM, this system is able to mass resolve more features with improved mass accuracy and therefore image more molecules within a single DESI MSI experiment, compared to other benchtop ToF instruments. The high mass resolution of the Xevo MRT MS allows for the differentiation of molecules close in mass, preventing the combining of spectral peaks which may lead to incorrect identifications or distributions being reported when using lower resolution mass spectrometers.

## Data Availability

Due to the size of the data, the data supporting the conclusion will be made available upon request.

## Supplementary Materials

The following supporting information can be downloaded at: https://www.mdpi.com/article/10.3390/metabo16060429/s1, Data S1: Extracted regions of interest and calculated correlations.

## Abbreviations

The following abbreviations are used in this manuscript:

MS	Mass Spectrometer
DESI	Desorption Electrospray Ionisation
LC/MS	Liquid Chromatography Mass Spectrometry
MRM	Multi Reaction Monitoring
EDC	Enhanced Duty Cycle
ToF	Time-of-Flight
FWHM	Full Width Half Maximum
PPM	Parts Per Million
PPB	Parts Per Billion
CLMC	Continuous Lock Mass Correction
RT	Room Temperature
API	Active Pharmaceutical Ingredient
XIC	Extracted Ion Chromatogram
BPI	Base Peak Intensity
TIC	Total Ion Count
PE	Phosphatidylethanolamine
PI	Phosphatidylinositol
PS	Phosphatidylserine
FA	Fatty Acid
CID	Collision Induced Disassociation
S/N	Signal to Noise
RGB	Red, Green, Blue

## References

[1] Takáts Z., Wiseman J.M., Gologan B. (2004). Cooks Mass spectrometry sampling under ambient conditions with desorption electrospray ionization. Science.

[2] Ifa D.R., Wu C., Ouyang Z., Cooks R.G. (2010). Desorption electrospray ionization and other ambient ionization methods: Current progress and preview. Analyst.

[3] Jackson A.U., Tata A., Wu C., Perry R.H., Haas G., West L., Cooks R.G. (2009). Direct analysis of Stevia leaves for diterpene glycosides by desorption electrospray ionization mass spectrometry. Analyst.

[4] Feider C.L., Elizondo N., Eberlin L.S. (2016). Ambient Ionization and FAIMS Mass Spectrometry for Enhanced Imaging of Multiply Charged Molecular Ions in Biological Tissues. Anal. Chem..

[5] Towers M.W., Karancsi T., Jones E.A., Pringle S.D., Claude E. (2018). Optimised Desorption Electrospray Ionisation Mass Spectrometry Imaging (DESI-MSI) for the Analysis of Proteins/Peptides Directly from Tissue Sections on a Travelling Wave Ion Mobility Q-ToF. J. Am. Soc. Mass Spectrom..

[6] Garza K.Y., Feider C.L., Klein D.R., Rosenberg J.A., Brodbelt J.S., Eberlin L.S. (2018). Desorption Electrospray Ionization Mass Spectrometry Imaging of Proteins Directly from Biological Tissue Sections. Anal. Chem..

[7] Zickuhr G.M., Um I.H., Laird A., Harrison D.J., Dickson A.L. (2024). DESI-MSI-guided exploration of metabolic-phenotypic relationships reveals a correlation between PI 38:3 and proliferating cells in clear cell renal cell carcinoma via single-section co-registration of multimodal imaging. Anal. Chem..

[8] Tillner J., Wu V., Jones E.A., Pringle S.D., Karancsi T., Dannhorn A., Veselkov K., McKenzie J.S., Takats Z. (2017). Faster, More Reproducible DESI-MS for Biological Tissue Imaging. J. Am. Soc. Mass Spectrom..

[9] Quartier J., Rao W., Slade S., Metral F., Lapteva M., Kalia Y.N. (2021). DESI-MS imaging to visualize spatial distribution of xenobiotics and endogenous lipids in the skin. Int. J. Pharm..

[10] Towers M.W., DeHoog R.J., Godfrey T.M., Towers L.A., Jones E.A., Ballantyne J.B., Suliburk J.W., Eberlin L.S. (2026). Development of Low-Flow High-Resolution Desorption Electrospray Ionization Mass Spectrometry Imaging. J. Am. Soc. Mass Spectrom..

[11] Claude E., Towers M., Lafont R., Wilson I.D., Plumb R.S. (2020). High Performance Thin-Layer Chromatography of Plant Ecdysteroids Coupled with Desorption Electrospray Ionisation–Ion Mobility–Time of Flight High Resolution Mass Spectrometry (HPTLC/DESI/IM/ToFMS). Chromatographia.

[12] Dill A.L., Eberlin L.S., Zheng C., Costa A.B., Ifa D.R., Cheng L., Masterson T.A., Koch M.O., Vitek O., Cooks R.G. (2010). Multivariate statistical differentiation of renal cell carcinomas based on lipidomic analysis by ambient ionization imaging mass spectrometry. Anal. Chem..

[13] Frisch K., Nielsen K.L., Hasselstrom M.J., Fink R., Rasmussen S.V., Johannsen M. (2024). Desorption Electrospray Ionization Mass Spectrometry Imaging of Powder-Treated Fingermarks on Forensic Gelatin Lifters and its Application for Separating Overlapping Fingermarks. Anal. Chem..

[14] Parrot D., Papazian S., Foil D., Tasdemir D. (2018). Imaging the Unimaginable: Desorption Electrospray Ionization—Imaging Mass Spectrometry (DESI-IMS) in Natural Product Research. Planta Med..

[15] Wiseman J.M., Ifa D.R., Zhu Y., Kissinger C.B., Manicke N.E., Kissinger P.T., Cooks R.G. (2008). Desorption electrospray ionization mass spectrometry: Imaging drugs and metabolites in tissues. Proc. Natl. Acad. Sci. USA.

[16] Ellis B.M., Fischer C.N., Martin L.B., Bachmann B.O., McLean J.A. (2019). Spatiochemically Profiling Microbial Interactions with Membrane Scaffolded Desorption Electrospray Ionization-Ion Mobility-Imaging Mass Spectrometry and Unsupervised Segmentation. Anal. Chem..

[17] Eberlin L.S., Ferreira C.R., Dill A.L., Ifa D.R., Cheng L., Cooks R.G. (2011). Nondestructive, Histologically Compatible Tissue Imaging by Desorption Electrospray Ionization Mass Spectrometry. ChemBioChem.

[18] Verenchikov A.N., Wildgoose J., Kirillov S.N., Vorobyev A.V., Makarov V.V., Gethings L.A., Tonge R.P., Daly M.E., Johnson W.J., Langridge J.I. (2026). A Novel Compact Multi-Reflecting Time-of-Flight Mass Spectrometer. J. Am. Soc. Mass Spectrom..

[19] Takács J., Hámori J. (1994). Developmental dynamics of Purkinje cells and dendritic spines in rat cerebellar cortex. J. Neurosci. Res..

